# Understanding what impacts on disclosing anal incontinence for women when comparing bowel-screening tools: a phenomenological study

**DOI:** 10.1186/s12905-019-0840-0

**Published:** 2019-11-21

**Authors:** Julie Tucker, Elizabeth Mary Ann Murphy, Mary Steen, Vicki L. Clifton

**Affiliations:** 1 Robinson Research Institute, School of Medicine, University of Adelaide, North Tce, Adelaide, Australia; 2 Departments of Surgery Lyell McEwin Hospital, Haydown Rd, Elizabeth Vale, South Australia Australia; 3 School of Nursing and Midwifery, University of South Australia City East Campus, Playford Building, Adelaide, SA 5000 Australia; 4 The Robinson Research Institute, School of Medicine, University of Adelaide, North Tce, Adelaide, Australia; 5 Mater Medical Research Institute, University of Queensland, Brisbane, Australia

**Keywords:** Anal, Incontinence, Women, Reproductive, Screening, Tools

## Abstract

**Background:**

There is limited research defining the true prevalence of anal incontinence (AI) in women of childbearing age. Understanding the limitations of the current assessment tools in the identification of AI is paramount for identifying the prevalence of AI and improving the care and management for women of childbearing age. The aim of this research was to explore and develop an understanding of women’s experiences in disclosing AI when completing a new bowel-screening questionnaire when compared to two established AI tools.

**Methods:**

A phenomenological qualitative research study was undertaken in a maternity setting in a large tertiary hospital. Parous women in the first trimester of a subsequent pregnancy were recruited to complete a specifically designed screening tool (BSQ), St Marks Faecal incontinence score (Vaizey) and Cleveland (Wexner) score. Qualitative semi-structured interviews were utilised to identify experiences in disclosing AI.

**Results:**

Women (*n* = 16, 22–42 years) with a history of anal incontinence either following the first birth (*n* = 12) or the second (*n* = 4) provided differing responses between the three assessment tools. All women answered the BSQ while the Vaizey and Wexner scores were more difficult to complete due to clinical language and participants level of comprehension. Women identified three major themes that were barriers for disclosing incontinence, which included social expectations, trusted space and confusion.

**Conclusion:**

There are barriers for disclosing AI in the pregnant and post-natal population, which can be improved with the use of an easy assessment tool. The BSQ may facilitate discussion on AI between the patient and health professional leading to earlier identification and improvement in short and long-term health outcomes.

## Background

Anal incontinence (AI) has a detrimental impact on quality of life. The cause of AI is multifactorial including direct/indirect trauma to muscle and nerves of the pelvic floor following vaginal births [1–4]. Compounding injury to the pelvic floor with subsequent birthing increases the risk of worsening symptoms of AI for women in both the short and long term [2, 5]. Evidence exists to support the use of bowel screening tools in the identification of AI within the pregnant and postnatal population [6–8]. Screening tools are adopted for research but limited in clinical practice as research identifies specialities involved in obstetrics and gynaecology rarely screen women for AI [9]. Importantly, there is no tool, routinely used to screen this at risk group, and the true prevalence of AI in women of reproductive age remains unknown.

Clinically derived bowel screening tools have been utilised in research to report AI in women in the late stages of pregnancy (8–65%), following birth (16–49%), gynaecology outpatients (16–28%), and in the general population (4.4%) [6, 10]. The wide variations in reporting may be a consequence of the definitions utilised to identify AI, the sample size, population studied, selective disclosure, the length and language comprehension of the questionnaire, how the tool was administered [8, 11–14]. Understanding the limitations of the current assessment tools in the identification of AI assists in defining true prevalence and improving the care and management for women of reproductive age.

Minimal research has explored the reasons for discordance between different screening tools from the afflicted person’s perspective. Research undertaken by Bartlett et al. [11] identified disagreement between two bowel screening tools, the Self-Administered Faecal Incontinence Questionnaire (SAFIQ) and the Cleveland Clinic Florida Faecal Incontinence Score (CCF-FI). Bartlett et al. [11] cited terminology and embarrassment were barriers to disclosure and suggested direct enquiry by a health professional utilising an AI questionnaire, in language that was easily understood could improve disclosure. Qualitative research by Tucker et al. [15] identified that women with a history of obstetric anal sphincter injury experienced barriers for disclosing AI and concur with these findings. Concurrent research identified active screening with assessment tools increased reporting of AI [8]. These findings instigated the development of a Bowel Screening Questionnaire (BSQ). The design and pilot testing of the BSQ included qualitative interviews with a group of symptomatic women. The aim of the qualitative research was to explore and develop an understanding of women’s experiences in disclosing AI when completing the BSQ and two established bowel assessment tools.

## Methods

Ethical approval was provided through the University of Adelaide Human Research Ethics Committee and the Human Research Ethics Committee (HREC/14/TQEHLMHMH/58). Research was undertaken between January 2015 and May 2017 and forms part of a larger research project. The research aimed to develop and validate the BSQ to identify AI in antenatal women attending a large tertiary hospital within a low socio-economic demographic area in South Australia. The antenatal triaging midwives invited parous women in their first trimester of a subsequent pregnancy with a previous history of AI to participate in the qualitative research. Nulliparous and asymptomatic women were not included.

Confidentiality was maintained for women who consented to the research. All texts were de-identified and information stored on a secure password protected universal serial bus. Women were aware that if the research caused any distress they could be referred to appropriate services or withdraw from the research without penalty. The main author (JT) undertook interviews. The Consolidated criteria for reporting qualitative research (COREQ) guidelines were adhered to for the research.

Interpretive phenomenology was adopted as a framework for this research [16]. A qualitative research method utilising semi-structured open-ended interviews, verbatim-transcribed text and journaling was used to explore and develop an understanding of women’s experiences of disclosing AI. Interviews were audio taped which enabled the researcher and women to immerse themselves within the conversations, and to record the realm of emotions and depth of the women’s individual experiences. Recruitment continued until no new themes were evident and saturation of data occurred at 16 women.

Research interviews were undertaken at a place and time that was convenient for the woman. Prior to the interview, women completed three bowel assessment tools including BSQ specifically developed by our team for the improved identification of AI. It was compared to the St Mark’s faecal incontinence score (Vaizey score) and Cleveland score (Wexner score) [17, 18]. The Vaizey and Wexner scores were included as they are frequently cited in research for this cohort [9, 19].

The Vaizey score is a validated tool and consists of two scoring systems with a five-point scale, which evaluates type and frequency of solid/liquid stool loss, flatus incontinence and impact on quality of life [18]. The Vaizey score is based on the Wexner score (non-validated score), the former including constipation and rectal urgency [19].The scoring system assesses symptoms over the last month and ranges from 0 (continent) to 24 (total incontinence). Both the Vaizey and Wexner scores are utilised in clinical studies and surgical therapies [19].The development of the BSQ occurred in consultation with health professionals and women with a history of AI. After review of questions, frequency scales and symptoms from establish tools for AI; the BSQ consisted of a symptom scale similar to the Vaizey and Wexner scores. The BSQ included six items prefaced with the statement “have you ever lost by accident?” included additional symptoms of staining, soiling, and request for referral (Table 1). A frequency scale measured symptoms and utilised a scale 0 (never) to four (daily) not unlike the Vaizey and Wexner scores. Previous research identified the variable nature of AI impacting on young women’s quality of life as such no timeframe was included [15, 20, 21].

**Table 1:** Bowel screening questionnaire (BSQ)

BSQ Qualitative interviews	Never	Rarely	Sometimes	Weekly	Daily
Answer the questions by placing a tick in the column	0	1	2	3	4
Have you ever lost by accident?					
Solid poo (Stool)					
Liquid poo (Stool)					
Wind (gas)					
Stained your underwear					
Soiled your underwear					
Do you need to rush to the toilet					
Total score /24 > 0 consider referral					
Would you like to be referred to a specialist Doctor?(Circle response)	Yes		No		

Interviews began by asking the woman to describe what it was like living with AI and to share what factors influenced or inhibited the disclosure of AI when using the three assessment tools. Initially there was uneasiness with the interview process through limited depth to responses and nonverbal body language. The style of the interview encouraged rapport and empathy through a conversational nature, with interviews typically lasting between 30 to 50 min. Women were able to seek assistance or clarification from the researcher in the completion of the tools.

### Data analysis

Transcribed verbatim texts, audio recordings and journal entries were analysed utilising Van Manen’s thematic analysis [16].The backwards and forwards process of the hermeneutic circle within the research process assisted in the reflection and uncovering the essence of women’s experiences. The repetitious reading and writing of data further developed meaning. Thematic analysis uncovered significant statements and led to the development of three themes and four sub-themes (Tables 2 and 3).

**Table 2:** Themes, sub themes and meanings

Theme	Sub theme	Meaning
Social expectation	Birthing process	Normal consequence, worse in subsequent delivery
	Keeping it hidden	Self-preservation, avoiding shame, waiting to be asked
Trusted space	Finding a voice	Safe environment to tell, Being safe, knowing someone understands, listen too, sensitive questions, help me understand the words
Confusion	Understanding	embarrassing words, defining words, capture variability

**Table 3:** Themes, sub themes and women’s statements

Theme	Sub theme	Example
Social expectation	Birthing process	“That’s what happens after a baby… I thought it didn’t matter” “It gets worse in your next pregnancy”
	Keeping it hidden	“Health professionals don’t ask and I feel horrible that I pass gas all the time” “I would tell if someone asked me” “ask me in words I understand”
Trusted space	Finding a voice	“use words that don’t make me feel any more disgusted in myself than I already do” “I need to feel safe to tell…” “How can I tell you I poo myself when I don’t know what the word stool is?”
Confusion	Understanding	“Telling a woman is easier… she’s been there and birthed” “Health professionals need to be sensitive when asking….I feel dirty and disgusted in myself already” “I don’t understand incontinence or urge…. What do they mean?” …what’s a plug?” “I don’t get symptoms all the time” “If you ask ….I would tell to get help”

## Results

Participating women (*n* = 16) were aged between 22 and 42 years and had a history of AI. All women identified English as first language; and were either Caucasian (*n* = 13, 81%) or Aboriginal (*n* = 3, 19%). Participants were predominately (75%) presenting in a second pregnancy, with 25% of women in their third pregnancy. Mode of birth included normal vaginal delivery (*n* = 13, 81%), forceps (*n* = 2, 13%) and caesarean section (*n* = 1, 6%).

Women who were symptomatic of AI following their principal birth accounted for 12 women (75%) with four women (25%) in their second pregnancy. Twelve women (75%) described a history of obstetric anal sphincter injury. Nine (75%) of these women reported the onset of AI following sphincter injury (Table 4).

**Table 4:** Demographic details of participants

Number *n* = 16	
Age, yrs., mean (range)(SD®)	31 (22–42) (7)
Ethnicity n (%)	
Caucasian	13 (81)
Aboriginal TSI	3 (19)
Parity n (%)	
2nd	12 (75)
3rd	4 (25)
Gestation weeks, mean (range)(SD)	22 (20–28)(3.5)
English 1st language n (%)	16 (100)
BMI^b^ mean (range)(SD)	30 (21–42)(7.5)
Mode of delivery n (%)	
Normal vaginal	13 (81%)
Forceps	2 (13%)
Caesarean section	1 (6%)
History OASIS ^a^n (%)	12 (75%)
Onset of AI post OASIS	9 (75%)
AI^c^ n (%)	
Following 1st birth	12 (75%)
Following 2nd pregnancy	4 (25%)

^a^Obstetric anal sphincter injury; ^b^body mass index;

^c^Anal incontinence®Standard deviation

### Initial interpretation of the screening tools

All women who participated in the qualitative interviews completed and provided a detailed account of disclosing AI using the assessment tools, the Vaizey score, Wexner score and BSQ. Women were unaware the BSQ had been newly designed to assess AI. Women identified a disparity in reporting between the three assessments tools, the BSQ easily completed and the Vaizey and Wexner were more difficult to complete due to clinical language and comprehension. The Vaizey and Wexner scores required the assistance of the researcher to understand aspects of the tools.

The Vaizey score was reported to be the most difficult to understand due to the clinical language, including incontinence, stool, plug and defecation. The variability of liquid, solid stool and flatus were assessed by a scoring system from zero to four for each symptom. However, women were critical that the Vaizey score reported symptoms in the last month. Additionally, the absence of a scale to identify the frequency of rectal urgency considered by women to underrepresent the variability of their symptoms.

Whilst the Wexner score included clinical language, women described the wording of this tool easier to understand and the preferred option to the Vaizey score. The Wexner score provided a frequency scale for symptoms with no timeframe. However, women detailed the exclusion of rectal urgency as a major limitation of the Wexner score as it was a predominant symptom for women.

The BSQ was the unanimously preferred tool, with women describing the language and tool design easy to understand when disclosing AI. A statement “have you lost by accident?” prefaced the BSQ and identified solid/liquid stools, flatus, urgency and staining and included a frequency scale 0 (never) to four (daily). Women stated a defined frequency scale for symptoms was preferred. Whilst the strengths and limitations of all tools were identified, women further described important additions in all tools. A frequency scale that identified a past and current history of solid/liquid stool, flatus, rectal urgency and staining was required.

### Deeper interpretation in disclosing AI

The disclosure of AI was reported as complex for most women. Understanding what the tool actually intended to ask influenced full disclosure. Women identified three major themes that described how they responded to disclosing AI with the three assessment tools. The themes included s*ocial expectations, trusted space and confusion* (Table 2 and 3).

### Social expectations

Social expectations were an overarching theme and included two sub themes *keeping it hidden* and the *birthing process*. Women within this research identified how this influenced the completion of the bowel assessment tools.

### Keeping it hidden

Keeping it hidden detailed how the social stigma surrounding AI limited disclosure. Young women identified the daily struggle of anxiety and despair in an attempt to maintain their dignity in society and coping with the consequences of the need to rush to the toilet and accidental loss of gas. They limited activities of daily living.

*“I feel anxious, the sound and smell is always on your mind, you don’t go out…I feel dirty” (Participant 13).*

*“It took a while for me to feel confident to tell someone, I was really embarrassed …..the urgency was bad but I had to tell someone eventually” (Participant 3).*

### Birthing process

All women commented on the negative impact of vaginal delivery on pelvic floor function, resulting a weakened pelvic floor and continence issues. There was resignation that the birth process and size of babies would have a negative impact on their pelvic floor, and as such, there was no reason to disclose symptoms. However, the concern for worsening AI was a reality with further birthing. Women stated that symptoms were often variable but worse in pregnancy. They were often not aware as to the clinical significance and under reported symptoms. Women described these concerns with the following statements,

*“Women aren’t aware of the importance of AI… it’s not seen as a problem because that comes with birthing” (Participant 1).*

*“It happens to all women…doesn’t it? So it’s not a problem, so why tell anyone” (Participant 8).*

*“I don’t get symptoms all the time, sometimes you get worse in pregnancy…. I worry for it (AI) will get worse in next pregnancy and birth. You don’t really want to be in nappies!” (Participant 16).*

### Trusted space

Participants outlined several key factors to improve disclosure. There was the need to be in a safe environment with those who identified with their situation enabling women to feel comfortable in *finding a voice*. There was a real sense of yearning for health professionals to ask questions related to continence, active screening by the health professional improved disclosure. Women recounted how the bowel assessment tools would help this process but the tool needed to be sensitive and use everyday words.

*“It’s never easy to tell…not in a busy place, it needs to be private and I need to trust the health professional. I cannot tell you if I do not understand what you are asking me…the words are confusing. The health professional helped me tell by asking questions differently…I don’t understand incontinence” (Participant 6).*

“*Health professionals need to ask especially for those of us who are from different cultural backgrounds….because I won’t tell otherwise” (Participant 13).*

*“I get embarrassed about my symptoms mostly urgency. I think health professionals need to ask about AI. Sometimes it’s easier telling a woman, she understands because she’s had a baby” (Participant 14).*

### Confusion

The theme *confusion* identified the disparity in reporting symptoms were largely a consequence of not understanding the questions. Women described the difficulty in understanding definitions, clinical language and inability of the tools to capture their symptoms of AI.

The women within this research study described the important role of the health professional in completing the screening tool and disclosing AI. The interpretation of questions by the women often resulted in confusion where women would respond with no symptoms if they did not understand the language or meaning.

*“What does incontinence mean? The words are technical; I do not know what they mean… So even if I do have urgency or staining I say I have no symptoms” (Participant 8).*

*“My symptoms are all over the place especially when I’m pregnant….the tools don’t see that? Simple words are needed…. tricky words like continence and defer need changing… who understands that?” (Participant 1).*

*“I need someone to help me understand the words; I don’t know incontinence, urgency, and plug. I get staining but is that liquid poo or is that different…I am very confused. I think it’s good to sit with the professional and discuss the questions (assessment tools), it’s hard otherwise to understand the words” (Participant 14).*

## Discussion

Disclosure of AI when utilising screening tools is reliant on multiple factors. Social expectations, role of the health professional, and how a screening tool is developed is pivotal in enabling or inhibiting disclosure of AI.

*Social expectations* were the overarching theme, which identified AI as a taboo health and wellbeing issue in many cultural settings. How we are socialised, will influence what illnesses we consider socially acceptable and either inhibit or promote health-seeking behaviour. The inability to conform to societal norms and the mere thought of discussing bodily functions often results in personal disgust and shame affecting disclosure [15, 22–24]. The physical and psychological impact of AI following obstetric anal sphincter injury is detailed by Tucker et al. [15] who identified the self-imposed social isolation, negative health-seeking behaviours and non-disclosure of AI [15]. These findings concur with our research findings, which identified the emotional complexity surrounding AI, stigma, embarrassment and personal disgust limited disclosure to health professionals and full disclosure by clinical screening tools. Embarrassment is reported as another key factor in the underreporting of AI within assessment tools [11].

Health seeking behaviour and disclosure relies on those afflicted with AI to have an understanding or knowledge of the problem [25]. Women within this research identified AI was a normal consequence of birthing and therefore described no reason to seek help or disclose AI. Importantly, whilst it was acknowledged there were concerns about worsening function in subsequent births disclosure was compounded as a result of pending social stigma. Additional research supported these findings, acknowledging nondisclosure was the result of the duality of the consequences of birthing and embarrassment associated with AI [6, 15, 23].

The role of the health professional in the initiation of questioning was pivotal in enabling or inhibiting disclosure of AI. Tucker et al. [15] concur with these findings further citing lack of enquiry by professionals was often viewed by women, as the clinician having limited knowledge or fear of client dissatisfaction in the assessment of continence status. Women from the current research identified with these conclusions noting a safe environment or, a *trusted space* and preference for female health professional facilitated the disclosure of AI. The partiality for female health professionals in genital and anal examinations is identified in the literature to facilitate patient centred communication, increased empathy and resultant reduction in embarrassment in reporting symptoms [15, 23, 26]. Whilst there is mixed debate as to the effectiveness of self-reported questionnaires and assessment tools versus clinician assisted discussion, the benefits of sensitive discussion with the latter have been shown to promote disclosure of AI [7, 11, 23]. Whilst this was evident within our findings, women identified a disparity in disclosure between the three assessments tools was a major limitation which focused on the design of the tool, and in particular language and comprehension.

Traditionally bowel screening and assessment tools objectively review type and frequency of AI from a clinicians point of view [19]. The limitations of clinically derived screening tools have been outlined previously [11, 14]. The confusion with terminology and the inability of the screening tools to capture symptoms was evident within our study. This is a concern for women as the underreporting of AI further marginalises them from adequate clinical care and management in current and subsequent pregnancies, potentially worsening quality of life [9, 15].

In order to disclose AI, clinical assessment tools needed to be easy to understand. A higher reading and comprehension level of health tools often results in misunderstanding [27]. Leonard reported there is a need to evaluate whether health literature is at an appropriate level for the intended population [27]. Findings from the current research identified women’s confusion with terms such as continence, defecation, stool and plug in utilising the Vaizey and Wexner scores. This may have been a result of the study population, who were recruited from a tertiary setting within a low socioeconomic demographical area. Confusion often resulted in no symptoms identified in reporting across two of the clinically derived assessment tools the Vaizey and Wexner scores. Preferred terms for incontinence included accidental leakage and is previously supported by Sung et al. [28] who identified AI did not reflect the patients perspective. Additionally research by Cotterill et al. [14] identified the importance of the patient’s perspective in the development of clinical tools and identified key areas not previously addressed. Women in the current research study identified the BSQ as the preferred assessment tool; because it did not contain any of the confusing terminology and each statement was prefaced with “*have you ever lost by accident”.* The acceptability of the BSQ may be due to the fact it was developed in consultation with women with a history of AI.

The current research identified the disclosure of symptoms often relied on the variability and impact of symptoms on quality of life with less frequent symptoms resulting in underreporting. Research undertaken by Bartlett et al. [11] supported these findings. The inability of the Vaizey and Wexner scores to capture the frequency of rectal urgency was cited as a limitation for this group of women. Previous research identified the shortcomings of assessment tools that do not incorporate the frequency of rectal urgency [19, 29]. Rectal urgency is associated with external anal sphincter injury and precursor of worsening symptoms across the lifespan. The ability of screening tools to identify the frequency of rectal urgency is important in the management of women of reproductive age.

Staining was an additional symptom women in our research identified. The symptom was variable but did result in considerable bother. Sung et al. [28] utilising qualitative research identified mucous loss as a consistent finding that required acknowledgement and inclusion in future frameworks on AI.

Women described the importance of a frequency scale from zero (no symptoms) to four (daily symptoms) to describe the variability of all symptoms. However, they noted limitations of the assessment tools, as they did not effectively identify the variability of symptoms across a longer period, in particular rectal urgency. These findings are consistent with previous research and promote the development of a screening tool which identifies a history of AI and current symptoms over the past 4 weeks [9].

### Strengths and limitations

Qualitative research findings are often limited in generalisability to a larger population. However, findings from this research may be applicable to research which aims to develop tools to identify AI in this population. The research method facilitated open disclosure and increased the richness of women’s stories but also allowed potential bias. Research bias was reduced through the invitation for women to take part by triaging antenatal midwives. The nature of the research question and the researcher’s role as a health professional may have influenced full disclosure. Despite this, the research methods facilitated open disclosure. The absence of a defined timeframe for the BSQ may be seen as a further limitation if utilised as a standalone tool. However, the aim of the BSQ was to be utilised in clinical practice to screen for AI and promote discussion of symptoms. Findings of the research-identified women wanted both historical and current symptoms identified by assessment tools and provides further information for future tool development.

## Conclusion

Findings of this research identify factors, which enabled and inhibited disclosure of AI utilising three bowel assessment tools. Women in this research identified the construction and development of screening and assessment tools should consider the appropriate language, comprehension of the tool, account for the variable nature of AI, the frequency of rectal urgency and staining. The BSQ was the preferred screening tools, as it was easy to understand and quick to utilise. However, women described the need for tools to include both historical and current symptom for AI. Whilst the findings are important for developing screening tools there is an urgent need by clinicians to understand the social stigma surrounding AI and the importance of using sensitive language in a safe environment to facilitate disclosure. Utilising screening tools like the BSQ in the pregnant and postnatal population will assist with disclosure and the early detection of AI that improves a woman’s short and long-term management and health outcomes.

## Data Availability

Data sharing is not applicable to this article as no datasets were generated during the current study.

## Abbreviations

AI: Anal incontinence
BSQ: Bowel screening questionnaire
Vaizey Score: St Marks Faecal incontinence score
